# Bioanalytical assay for the quantification of rucaparib in rat plasma using UPLC-MS/MS: development, and validation for interaction with myricetin

**DOI:** 10.3389/fphar.2025.1576131

**Published:** 2025-05-26

**Authors:** Jingjing Nie, Hailun Xia, Jie Chen, Jun Wu, Jinming Yang, Xuegu Xu, Congrong Tang

**Affiliations:** ^1^ Department of Pharmacy, The Third Affiliated Hospital of Wenzhou Medical University, Wenzhou, Zhejiang, China; ^2^ Department of Pharmacy, The First Affiliated Hospital of Wenzhou Medical University, Wenzhou, Zhejiang, China; ^3^ Department of Pharmacy, The Eye Hospital of Wenzhou Medical University, Wenzhou, Zhejiang, China

**Keywords:** rucaparib, myricetin, UPLC-MS/MS, rat plasma, pharmacokinetics

## Abstract

Rucaparib is used to treat ovarian cancer patients with BRCA gene mutations. Myricetin, a flavonol that strongly inhibits CYP450, is widely found in natural plants and has some anticancer properties, with the potential for combination use. However, there is no report on the interaction between myricetin and rucaparib. Therefore, an ultra performance liquid chromatography tandem mass spectrometry (UPLC-MS/MS) detection approach with high selectivity, reproducibility, sensitivity, and stability was established, which was used to explore the effect of myricetin on rucaparib metabolism in rats. In this study, acetonitrile was used as the protein precipitant, and fuzuloparib was used as the internal standard (IS). Method validation followed the bioanalytical method validation criteria outlined by the FDA. A good linear range was achieved in the range of 2.0–500 ng/mL. Intra-day and inter-day precision (RSD%) for rucaparib were both less than 7.1%, and accuracy (RE%) ranged from −1.2%–10.9%. Matrix effects were observed in 89.8%–99.7% with recovery exceeding 96.1%. The results of the drug-drug interaction (DDI) study showed that myricetin had no significant effect on the pharmacokinetic parameters of rucaparib, which indicating that the clinician did not need to adjust the dosage of rucaparib when it was used in combination. The UPLC-MS/MS method developed in this study was successfully used for the determination of the plasma concentrations of rucaparib orally administered in rats, which provided a reference for DDI studies and clinical pharmacokinetic studies of rucaparib.

## 1 Introduction

Rucaparib, an oral poly ADP-ribose polymerase (PARP) inhibitor, was received FDA (Food and Drug Administration) approval for maintenance treatment in adult patients with recurrent epithelial ovarian, primary peritoneal carcinomas, or fallopian tube carcinomas who possess a harmful BRCA1 or BRCA2 mutation (germline and somatic types are included) (Coleman et al., 2017; Oza et al., 2017). The most common adverse events with rucaparib are fatigue, nausea, and anemia or decreased hemoglobin, which can be mitigated by interrupting treatment or reducing the dose (Fizazi et al., 2023). In studies of the cytochrome P450 (CYP450) enzyme family, rucaparib belongs to the CYP2D6 substrate and is mainly metabolized by CYP2D6 and weakly metabolized by CYP1A2 and CYP3A4 (Liao et al., 2020).

The accumulation of drugs in the body has been demonstrated to increase the risk of adverse drug reactions. The process of drug absorption, distribution, metabolism, and excretion is often significantly impacted by plasma drug concentrations (Fan and de Lannoy, 2014). CYP450 enzymes play a pivotal role in drug metabolism (Almazroo et al., 2017; Zhao et al., 2021). Inhibition of these enzymes by CYP450 enzyme inhibitors is a primary cause of increased drug concentrations. It is a common practice among patients to utilize traditional Chinese medicine as a complementary treatment for their ailments. This approach is undertaken with the objective of mitigating complications and enhancing the quality of life (Sarris, 2018; Yeung et al., 2018). The significant impact of CYP450 enzyme inhibitors on plasma drug concentrations has been well-documented; however, the potential metabolic inhibition of herbal components has been overlooked. A substantial body of recent studies has reported on the effects of herbal components on drug concentrations. Among these components, myricetin has been shown to possess a significant inhibitory effect on drug metabolism (Fugh-Berman, 2000; Sabiu and Idowu, 2022; Ye et al., 2024). However, the results of an animal study demonstrated that myricetin did not lead to an accumulation of drug concentrations (Chen et al., 2024). Myricetin exhibits different effects on various substrates, and further investigation is necessary to ascertain whether it exerts an inhibitory effect on rucaparib.

Myricetin was firstly extracted from the bark of the prune tree in 1896 and is a flavonol compound (Song et al., 2021). Recent studies have shown that myricetin possesses various pharmacological activities, such as anti-inflammatory, antitumor, antibacterial, antiviral, and anti-obesity effects (Imran et al., 2021). It also exerts cardiovascular protection, protects against neurological damage, and safeguards the liver against potential injuries (Song et al., 2021; Chen et al., 2025). European countries developed and marketed health products containing myricetin owing to its antioxidant function and cholesterol-lowering effect. Due to its numerous pharmacological activities, myricetin has become increasingly popular among the public. Recent clinical studies have also reported a chemopreventive effect of myricetin on cell proliferation, reducing the risk of prostate and ovarian cancer (Gates et al., 2007; Geybels et al., 2013; Devi et al., 2015). It was reported that myricetin could inhibit CYP3A4/3A2, CYP2D6/2D1, CYP2C9/2C11, and CYP2B1 through different mechanisms *in vitro* (Lou et al., 2019). According to Okan et al., the average American consumes about 189.7 mg of flavonoids and 12.9 mg of flavonols per day; however, those who regularly consume garlic or black tea have a much higher flavonol intake than 12.9 mg (Okan et al., 2015). Myricetin intake leads to the inhibition of enzymes such as CYP2D6, CYP3A4, which can introduce uncertainty into the metabolism of certain drugs.

As observed from the drug metabolism, myricetin may affect the metabolism of rucaparib and increase its exposure *in vivo*. When the plasma concentration of rucaparib is suboptimal, it can trigger tumor recurrence and metastasis. Conversely, an elevated plasma concentration of rucaparib is associated with a heightened risk of severe adverse reactions, including urinary tract infections, liver function abnormalities, and potentially organ failure (Xiao et al., 2019; Grechko et al., 2021). Therefore, it is necessary to investigate the effect of myricetin on rucaparib plasma exposure to clarify the risk of drug interactions occurring and provide data to support rational clinical use.

To the best of our knowledge, two analytical methods for the determination of rucaparib with another PARP inhibitors in plasma using LC-MS/MS had been established to date (Bruin et al., 2020; Canil et al., 2023). However, these methods have long analysis time (4.5 min) and low sensitivity (50 ng/mL), which cannot meet the requirements of rapid and sensitive detection. Therefore, a rapid, sensitive and accurate ultra performance liquid chromatography tandem mass spectrometry (UPLC-MS/MS) method was developed in this study. The total run time was 2 min and the lower limit of quantification (LLOQ) was 2.0 ng/mL. The investigation further encompassed the alteration in plasma exposure of rucaparib in the presence of myricetin, along with the validation of the UPLC-MS/MS method. This comprehensive approach was undertaken to elucidate the underlying mechanisms and provide actionable insights into the clinical co-administration of these agents.

## 2 Experimental

### 2.1 Chemicals and reagents

Reference standards of fuzuloparib (used as the internal standard, IS; purity > 98%) and rucaparib (purity > 98%) were obtained from Beijing sunflower and technology development Co., Ltd. (Beijing, China). The analytical-grade formic acid, along with chromatographically pure acetonitrile and methanol, were sourced from Merck Company (Darmstadt, Germany). Ultrapure water was obtained through purification using Millipore’s Milli-Q ultrapure water system (Bedford, United States).

### 2.2 UPLC-MS/MS conditions

An ultra performance liquid chromatography system (Waters Corp., United States) was used to perform the chromatographic separation. It was separated by a mobile phase of 0.1% formic acid in water (A) and acetonitrile (B) at 40°C using an Acquity UPLC BEH C18 column (2.1 mm × 50 mm, 1.7 μm, Waters). The procedure was presented at a flow rate of 0.4 mL/min as follows: initially, 90% A was maintained from 0 to 0.5 min, then it was linearly decreased to 10% A (0.5–1.0 min), and 10% A was maintained from 1.0 to 1.4 min. Subsequently, the proportion of A was rose linearly to 90% (1.4–1.5 min), and maintained at 90% for 0.5 min. The injection volume for sample analysis was fixed at 2.0 µL.

**FIGURE 1:**
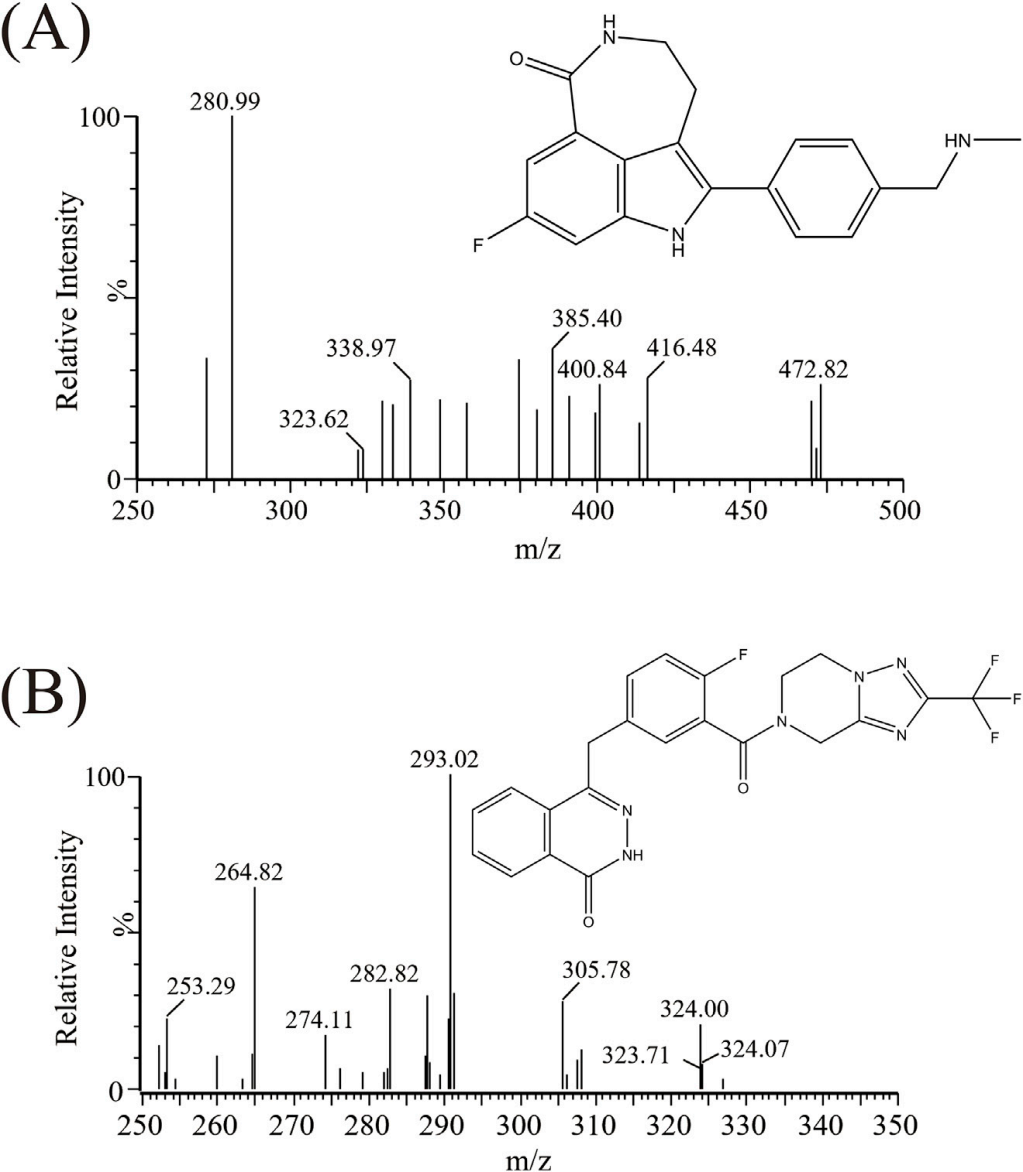
The mass spectra and chemical structures of rucaparib **(A)** and fuzuloparib **(B)**.

Triple quadrupole mass spectrometry (Waters Corp., United States) was utilized for detection. The mass spectrometry parameters of rucaparib and IS were as follows: cone voltages of 10 V and 30 V, and collision energies of 15 eV and 25 eV, respectively. The mass spectrometry (*m/z* 324.00 → 293.02 for rucaparib, *m/z* 472.82 → 280.99 for IS) was performed in positive ionization, as illustrated in Figure 1 and Table 1.

**TABLE 1:** Specific mass spectrometric parameter and retention time (RT) for rucaparib and IS, including cone voltage (CV), and collision energy (CE).

Analytes	Precursor ion	Product ion	CV (V)	CE (eV)	RT (min)
Rucaparib	324.00	293.02	10	15	1.14
IS	472.82	280.99	30	25	1.32

### 2.3 Formulation of working solutions and quality control samples (QCs)

The working solutions of rucaparib were achieved by diluting the stock solution (1.0 mg/mL) with methanol. Methanol was also used to prepare the IS working solution, which was set at a concentration of 200 ng/mL. Calibrated curve and quality control (QC) points were obtained by adding the appropriate rucaparib working solution (10 µL) to blank rat plasma (90 µL). The calibration curve has eight points with a concentration range of 2.0–500 ng/mL. Concentrations at four QC levels of 2.0, 5.0, 200, and 400 ng/mL were respectively corresponded to the LLOQ, low (LQC), medium (MQC), and high (HQC) concentrations, respectively. The samples and solutions were preserved in a refrigerator set at −80°C.

### 2.4 Sample processing

Before processing, the plasma samples were moved from −80°C to room temperature and thawed thoroughly. Subsequently, 100 µL of plasma and 10 µL of IS working solution were added to a 1.5 mL centrifuge tube, along with 300 µL of acetonitrile to precipitate the proteins. The mixture was centrifuged at 13,000 g for 10 min after being vortexed completely, and then 100 µL supernatant was transferred to autosampler vial for UPLC-MS/MS investigation.

### 2.5 Method validation method validation

#### 2.5.1 Selectivity

For selectivity, it refers to the capability of an assay to differentiate and accurately quantify the target compounds. Impurities were determined to be non-interfering with rucaparib by comparing the results from six different rat blank plasma, blank plasma containing standards (rucaparib 2.0 ng/mL) and real plasma samples collected after oral medication.

#### 2.5.2 Sensitivity and linearity

By measuring eight plasma samples at different concentrations (2.0–500 ng/mL) over 3 days, a standard curve was generated. The linear regression was performed by plotting the ratios of the peak area of rucaparib to IS (Y) versus the nominal concentrations of rucaparib spiked into rat plasma (X), and the coefficient of determination (*r* ^*2*^) was employed to evaluate the linear regression. The LLOQ was represented as the lowest rucaparib concentration measurable on the standard curve with acceptable precision and accuracy (accuracy within ±20%, precision ≤20%).

#### 2.5.3 Accuracy and precision

Determination of three different levels (5.0, 200, and 400 ng/mL) of QCs over 3 days was needed to determine accuracy and precision (n = 5). For the test results, the relative error (RE%) and relative standard deviation (RSD%) should be investigated to determine if they fell within the stated limits (RE within ±15%, RSD < 15%). These metrics could be used for assessing the systematic and random errors of the method.

#### 2.5.4 Extraction recovery and matrix effect

Extraction recoveries and matrix effects were examined using blank plasma from various rats at QC levels (5.0, 200, and 400 ng/mL). The evaluation of matrix effect was conducted by comparing the response of extracted plasma added rucaparib with that in a pure solution (n = 5). The recovery of the current sample preparation method was investigated by contrasting the peak area ratios before and after extraction.

#### 2.5.5 Stability

The stability experiment was conducted at three different QCs (5.0, 200, and 400 ng/mL). It was carried out under various storage conditions, with each condition having five replicates (n = 5). These storage conditions encompassed stability within the analyzer (lasting for 4 h at 10°C), long-term storage (for 3 weeks at −80°C), short-term storage (for 3 h at room temperature) and freeze-thaw cycles (conducted three times).

### 2.6 Drug-drug interaction (DDI) studies

Twelve Sprague-Dawley (SD) male rats were provided with an appropriate environment with unrestricted water and food for seven consecutive days prior to the experiment. The day prior to the study, the rats underwent a 12-h period of fasting while being allowed unfettered access to water. Subsequently, they were randomly assigned to two groups (n = 6). Group A was received an oral dose of rucaparib at 20 mg/kg, while Group B was received an oral administration of rucaparib at 20 mg/kg along with myricetin at 50 mg/kg. The doses of rucaparib (Augustine et al., 2019) and myricetin (Ye et al., 2024) administered were determined based on prior literatures. Rucaparib was formulated in corn oil with myricetin prepared in 0.5% carboxymethylcellulose sodium (CMC-Na) solution. Group B rats were administered a single gavage of 50 mg/kg of myricetin, while Group A rats were received an equivalent volume of CMC-Na solution. Subsequently, at the 30 min mark, both groups were administered a single dose of 20 mg/kg of rucaparib. Before dosing (designated as 0 h) and at 1.5, 3, 4, 5, 6, 8, 12, 24 and 36 h post-dosing, 0.3 mL of plasma samples were drawn from the caudal vein. These samples were collected into heparin-containing tubes and then centrifuged at 8,000 rpm at 4°C for 10 min. Afterwards, the plasma was transferred to new tubes and preserved at −80°C for further analysis.

The DAS 3.0 software was used to calculate the pharmacokinetic parameters of rucaparib using a non-compartmental model. The key pharmacokinetic parameters of the two groups were analyzed using an independent samples *t*-test in SPSS 24.0. The *p*-value of less than 0.05 indicated a significant difference between the two groups. Mean plasma concentration-time curves were generated with GraphPad Prism 9.0 software (GraphPad Software Inc., California, United States).

## 3 Results and discussions

### 3.1 Method validation

#### 3.1.1 Selectivity

**FIGURE 2:**
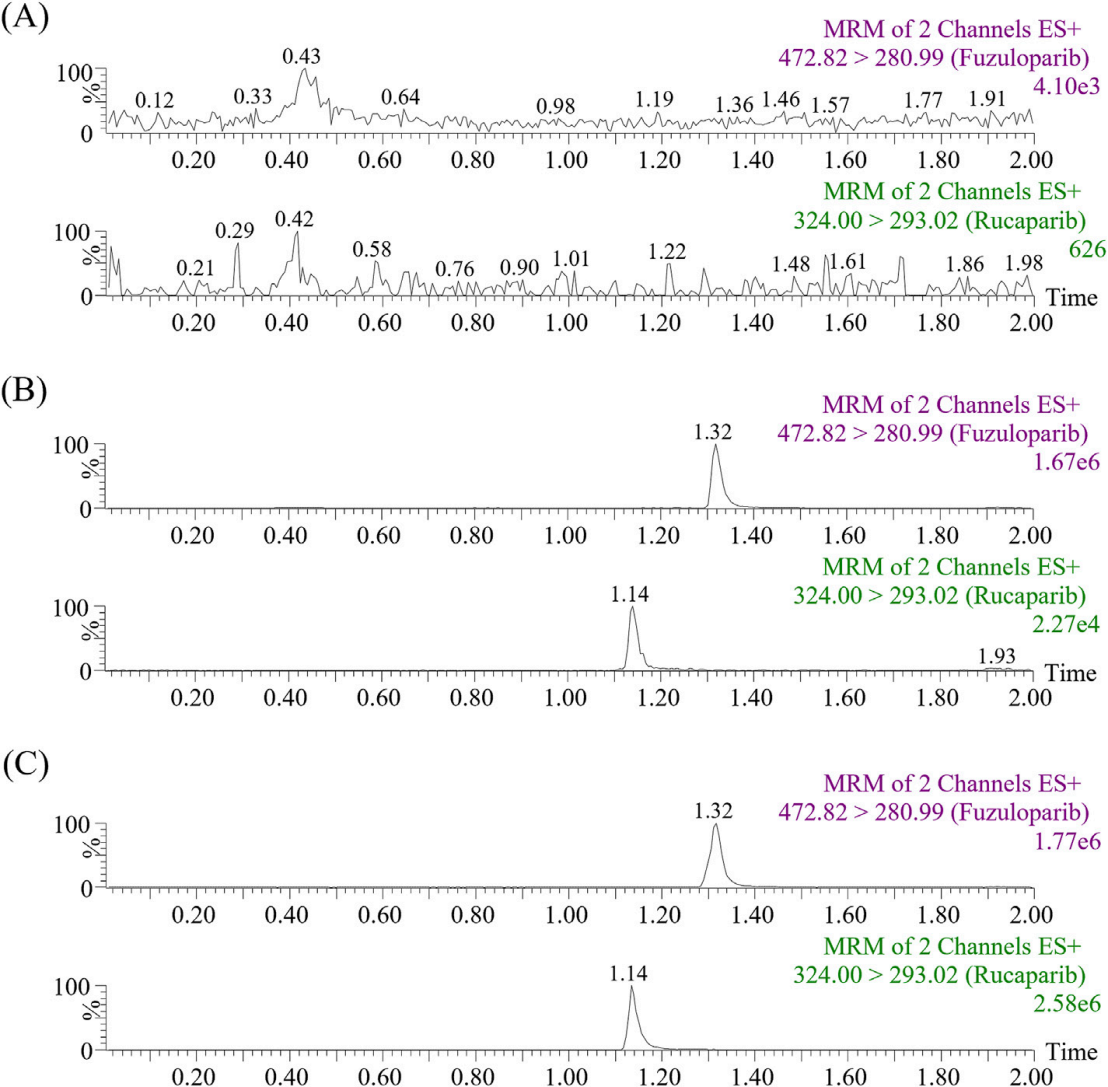
Representative MRM chromatograms of fuzuloparib (IS) and rucaparib in samples: blank plasma **(A)**, blank plasma spiked with standard solutions **(B)** and real plasma sample collected from a rat oral administration of 20 mg/kg rucaparib only **(C)**.

Figure 2 exhibited the chromatograms of blank plasma, blank plasma containing standard substances, and real plasma samples from rats treated orally with rucaparib. As presented, neither endogenous substances nor commonly used chemicals caused any interference with the target peaks. The relative retention times of rucaparib and IS were approximately 1.14 and 1.32 min, respectively.

#### 3.1.2 Linearity of calibration curve and LLOQ

In the range of 2.0–500 ng/mL, the fitted calibration curve for rucaparib in rat plasma showed good linearity, with a coefficient of determination (*r* ^*2*^) higher than 0.99. The typical regression equation shown in Table 2 was Y = 0.0112139*X − 0.00376457. The LLOQ of the developed method was 2.0 ng/mL. The RSD was <7.1%, and the RE ranged from 10.0% to 10.9%, respectively. According to the FDA guideline, these values fell within the acceptable range of ±20% (as shown in Table 3).

**TABLE 2:** Calibration curves for the analysis of rucaparib in SD rat plasma.

Analyte	Regression equation	*r* ^*2*^	Linear range (ng/mL)	LLOQ (ng/mL)
Rucaparib	y = 0.0112139x − 0.00376457	0.998	2.0–500	2.0

**TABLE 3:** The accuracy and precision of rucaparib in SD rat plasma (n = 5).

Analyte	Concentration (ng/mL)	Intra-day		Inter-day	
		RSD%	RE%	RSD%	RE%
Rucaparib	2.0	3.9	10.9	7.1	10.0
	5.0	1.2	0.0	2.5	−1.2
	200	2.3	2.1	2.5	0.7
	400	1.9	−0.3	3.4	3.1

#### 3.1.3 Accuracy and precision

Intra- and inter-day precision and accuracy were measured and calculated for the three concentration levels of QCs as well as the LLOQ, and were expressed as RE% and RSD%. The intra-day RSD% exhibited a value lower than 2.3%, with the RE% values distributed in the interval of −0.3%–2.1%. Meanwhile, the inter-day RSD% was less than 3.4%, and the RE% values ranged from −1.2% to 3.1% (Table 3). As the QC results met FDA guidelines, it was evident that the method demonstrated excellent accuracy and reproducibility.

#### 3.1.4 Recovery and matrix effect

The extraction recoveries and matrix effects of the QCs were presented in Table 4. With respect to the concentrations of 5.0, 200, and 400 ng/mL, the mean extraction recoveries were amounted to 98.9%, 96.1%, and 100.3%, respectively. Meanwhile, the matrix effects were 99.7%, 89.8%, and 94.7%, respectively. Based on the QC results, it was evident that the method was reliable and precise for quantifying rucaparib in biological samples.

**TABLE 4:** Recovery and matrix effect of rucaparib in SD rat plasma (n = 5).

Analyte	Concentration (ng/mL)	Recovery (%)		Matrix effect (%)	
		Mean ± SD	RSD (%)	Mean ± SD	RSD (%)
Rucaparib	5.0	98.9 ± 4.5	4.6	99.7 ± 4.2	4.2
	200	96.1 ± 1.8	1.8	89.8 ± 7.8	8.7
	400	100.3 ± 4.5	4.5	94.7 ± 8.8	9.3

#### 3.1.5 Stability

The stability of rucaparib in rat plasma was examined under four distinct storage conditions. These included short-term storage (lasting 3 h at room temperature), exposure to three freeze-thaw cycles (transitioning from −80°C to room temperature), long-term storage (at −80°C for 21 days), and a 4 h placement in an autosampler maintained at 10°C. As presented in Table 5, regardless of the storage conditions, the analyte showed stability with minor fluctuations, indicated by an RSD of less than 15%.

**TABLE 5:** Stability results of rucaparib in plasma under different conditions (n = 5).

Analyte	Added (ng/mL)	Room temperature, 3 h		Autosampler 10°C, 4 h		Three freeze-thaw		−80°C, 3 weeks	
		RSD (%)	RE (%)	RSD (%)	RE (%)	RSD (%)	RE (%)	RSD (%)	RE (%)
Rucaparib	5.0	4.2	−4.2	1.9	1.5	5.2	−10.1	4.3	5.7
	200	3.7	−11.5	3.2	1.4	1.7	−1.6	5.0	9.2
	400	5.0	−3.1	1.9	8.1	4.1	−11.6	6.7	1.4

### 3.2 DDI study

**FIGURE 3:**
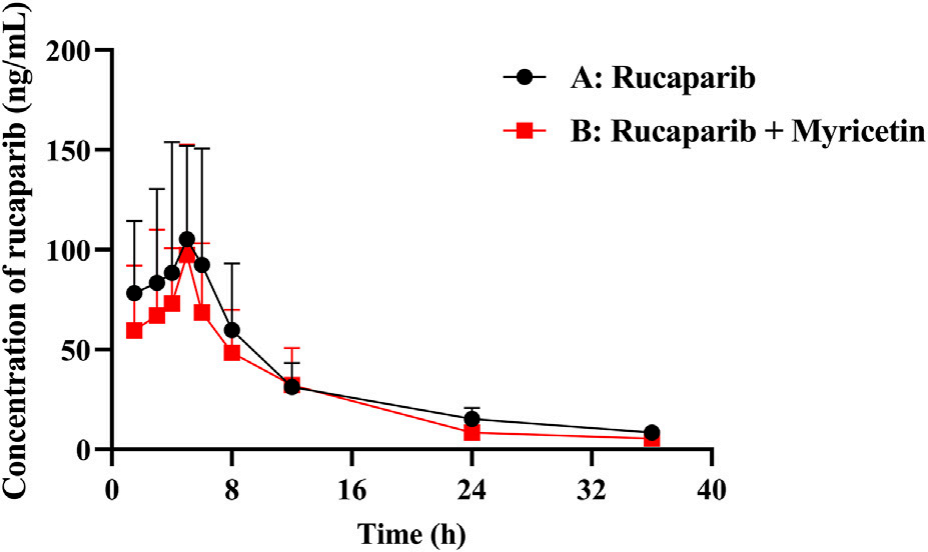
Mean plasma concentration–time curve of rucaparib in rats. (n = 6, Mean ± SD). (Group A: 20 mg/kg rucaparib dosed orally alone; Group B: 20 mg/kg rucaparib and 50 mg/kg myricetin dosed orally).

The mean plasma concentration profiles of Group A (20 mg/kg rucaparib only) and B (20 mg/kg rucaparib and 50 mg/kg myricetin) were summarized in Figure 3. The main pharmacokinetic parameters were presented in Table 6. The results showed that myricetin decreased the AUC_(0-t)_, AUC_(0-∞)_, t_1/2_ and C_max_ of rucaparib by 19.3%, 21.5%, 19.3% and 17.2%, respectively, while CL_z/F_ was increased by 16.3%. However, pharmacokinetic parameters were not statistically different between the two groups.

**TABLE 6:** The main pharmacokinetic parameters of rucaparib in two rat groups (group A: 20 mg/kg rucaparib; group B: 20 mg/kg rucaparib and 50 mg/kg myricetin) (n = 5, Mean ± SD).

Parameters	Rucaparib	Rucaparib + myricetin
AUC_(0→t)_ (ng/mL·h)	1,217.43 ± 502.63	982.32 ± 259.01
AUC_(0→∞)_ (ng/mL·h)	1,324.14 ± 487.13	1,038.99 ± 270.77
t_1/2_ (h)	10.88 ± 3.41	8.78 ± 3.00
T_max_ (h)	4.25 ± 1.78	5.67 ± 3.20
CL_z/F_ (L/h/kg)	17.62 ± 8.82	20.50 ± 5.86
C_max_ (ng/mL)	125.87 ± 58.53	104.18 ± 50.98

Compared with the Group Rucaparib alone. AUC, area under the plasma concentration-time curve; t_1/2_, elimination half time; T_max_, peak time; CL_z/F_, plasma clearance; C_max_, maximum plasma concentration.

## 4 Discussion

Rucaparib is a PARP inhibitor with inhibitory effect on BRCA gene mutations, and can be used in patients with BRCA gene mutations in ovarian cancer to prolong progression-free survival with a high safety profile (Dal Molin et al., 2018; Slade, 2020). It can be surmised that rucaparib will have a wider use in the future as clinical data are continued to be improved. However, there are few DDI studies on rucaparib. As reported by the FDA, rucaparib is primarily metabolized by CYP2D6 and weakly metabolized by CYP1A2 and CYP3A4 (FDA, 2016).

Myricetin, a flavonol widely found in plants, has anticancer, anti-inflammatory, and cardiovascular properties, and has the potential to be used as a food additive or herbal medicine in combination with rucaparib (Zhang et al., 2021; Marrero et al., 2022). However, myricetin can cause potential adverse drug events by inhibiting CYP3A4 causing increased drug exposure (Li et al., 2011; Ye et al., 2024). Previous study has reported some inhibition of CYP2D6 and CYP3A by myricetin (Lou et al., 2019). Rucaparib is primarily metabolized by CYP2D6 and to a lesser extent by CYP3A4. Given the potential for combination use of these two drugs, the risk of potential DDI should be assessed.

In this experiment, a highly selective, reproducible, sensitive, and stable UPLC-MS/MS-based assay was developed for the determination of rucaparib in rat plasma. Our results showed that this method exhibited good linearity over the concentration range of 2.0–500 ng/mL for rucaparib, with inter- and intra-batch precision and accuracy in accordance with the standard requirements, low residue levels and no matrix effects. These results demonstrated the validity of the current method for the detection of rucaparib concentrations in plasma.

Results of *in vivo* pharmacokinetic assays showed that myricetin had no significant effect on plasma exposure to rucaparib. One paper reported that myricetin inhibited CYP2D6 metabolism with the half-maximal inhibitory concentrations value of 57 μM, which is a weak inhibition of metabolism (Lee et al., 2012). Although myricetin inhibited CYP2D6, the weak inhibition was unable to significantly affect pharmacokinetic parameters. In addition, myricetin has low bioavailability and may not produce effective inhibitory concentrations *in vivo* (Ross and Kasum, 2002). The pharmacokinetics of rucaparib remain largely unaffected when co-administered with 50 mg/kg of myricetin, and the risk of potential DDI is minimal. In instances where higher doses of myricetin are utilized in combination, a re-evaluation of the potential risks is imperative. A limitation of our experiment was the single administration, which did not take into account the long-term co-administration of the drug. Furthermore, clinicians must consider interspecies differences when co-administering drugs.

In summary, an UPLC-MS/MS method had been developed for the precise and accurate detection of the concentration of rucaparib. The analytical method had been demonstrated to possess the capability of rapidly detecting the concentration of rucaparib with high sensitivity and accuracy, thereby providing an alternative clinical method for detecting drug concentration. Furthermore, the analytical method was successfully employed for the quantitative detection of rucaparib in combination with myricetin, which may facilitate future DDI studies. In conclusion, myricetin exhibited no substantial impact on the plasma exposure of rucaparib. The probability of DDI was minimal when these two medications were administered concomitantly.

## 5 Conclusion

For the first time, an UPLC-MS/MS method with high reliability and sensitivity was established in the current study for the measurement of rucaparib in rat plasma and validated according to FDA standards. In addition, it was used in a DDI study of rucaparib in rats. No significant changes in the pharmacokinetic parameters of rucaparib were observed after administration of 50 mg/kg of myricetin to rats, suggesting that no dose adjustment was required for the administration of rucaparib in combination with myricetin. However, considering the limitations of interspecies differences, further studies are needed to clarify the results.

## Data Availability

The original contributions presented in the study are included in the article/supplementary material, further inquiries can be directed to the corresponding author.
